# Supplementary material for: Cancer-Related Neuropathic Pain, Chemotherapy-Induced Peripheral Neuropathy and Cognitive Decline in a 5-Year Prospective Study of Patients with Breast Cancer—NEON-BC
Source: Healthcare (Basel). 2023 Dec 9;11(24):3132. doi: 10.3390/healthcare11243132 (PMC10743065; doi:10.3390/healthcare11243132)
Supplement: Supplementary file 1 [file healthcare-11-03132-s001.zip › healthcare-2705485-supplementary.pdf]

Neuropathic pain, chemotherapy-induced peripheral neuropa-thy and cognitive decline in a 5-year prospective study of patients with breast cancer – NEON-BC.

Susana Pereira, Natália Araújo, Filipa Fontes, Luisa Lopes-Conceição, Teresa Dias, Augusto Ferreira, Samantha Morais, Vítor Tedim Cruz, and Nuno Lunet

Supplementary material

Table S1. Cancer treatments received during the five years after diagnosis of breast cancer.

	Cancer treatments received		
	During the 1st year after diagnosis n (%)	Between the 1st and the 3rd year after diagnosis n (%)	Between the 3rd and the 5th year after diagnosis n (%)
Breast surgery			
Mastectomy	212 (45.9)	-	-
Mastectomy + breast-reconstruction	15 (3.2)	-	-
Breast-conserving	235 (50.9)	-	-
Breast reconstruction	-	26 (5.6)	33 (7.1)
Breast-conserving surgery for a contra-lateral breast cancer	-	1 (0.2)	3 (0.6)
Total mastectomy for a contra-lateral breast cancer	-	-	1 (0.2)
Axillary surgery[a]			
Sentinel lymph node biopsy	295 (65.9)	-	-
Axillary lymph node dissection	153 (34.1)	1 (0.2)	1 (0.2)
Metasectomy			
Hepatic metastasectomy	-	1 (0.2)	1 (0.2)
Cerebral metastasectomy	-	1 (0.2)	-
Chemotherapy			
Timing			
Neo-adjuvant	30 (10.8)	-	-
Adjuvant	249 (89.2)	-	-
For a recurrence or another primary cancer	-	5 (1.1)	10 (2.2)
Drugs			
Doxorubicin + cyclophosphamide	56 (20.1)	-	-
Doxorubicin + cyclophosphamide + docetaxel	29 (10.4)	-	-
Doxorubicin + cyclophosphamide + paclitaxel	1 (0.4)	-	-
Cyclophosphamide + docetaxel	2 (0.7)	-	-
Carboplatin + docetaxel	1 (0.4)	-	-
5-FU + epirubicin + cyclophosphamide	23 (8.3)	-	-
5-FU + epirubicin + cyclophosphamide + docetaxel	165 (59.4)	-	1 (0.1)
5-FU + cyclophosphamide + methotrexate	1 (0.4)	-	-
Capecitabine	-	2 (0.9)	3 (0.3)
Docetaxel	-	-	1 (0.1)
Paclitaxel	-	3 (0.8)	5 (0.5)
Vinorelbine	-	-	2 (0.2)
Carboplatin	-	-	1 (0.1)
Gemcitabine	-	-	2 (0.2)
Epirubicin	-	-	1 (0.1)

Rituximab + cyclophosphamide + doxorubicin + vincristine	-	1 (0.5)	-
Radiotherapy (chest, axillary and/or supraclavicular)	341 (73.8)	3 (0.7)	4 (0.9)
Endocrine therapy	388 (84.0)	385 (83.3)	379 (82.0)
Other systemic treatments			
Trastuzumab	61 (13.2)	-	2 (0.4)
Pertuzumab	-	-	1 (0.2)
Lapatinib	-	1 (0.2)	-

[a] Patients who had both axillary lymph node dissection and sentinel lymph node biopsy are reported as axillary lymph node dissection; N<462, because 14 patients only performed breast surgery.

Table S2. Neurological conditions identified at the baseline neurological exam.

**Neurological condition**	**n**
Low cognitive performance [a]	65
Migraine [b]	41
Tremor	19
Tension headache [c]	8
Diabetic Polineuropathy	3
Polymyelitis sequelae	3
Carpal tunnel syndrome	3
Amaurosis	2
Cerebrovascular disease	2
Epilepsy	2
Trigeminal neuralgia	2
Facial paralysis	2
Atrophy and loss of strength in the left arm	1
Mild left hemiparesis sequelae of meningitis	1
L5-S1 hernia	1
Left sciatic nerve injury	1
Tomaculous neuropathy	1
Parkinson' disease	1
Transient ischemic attack	1

[a] All cases were considered as probably related with cancer. Cognitive performance was assessed with the Montreal Cognitive Assessment, using age- and education-specific norms.[1]

[b] One case of migraine with aura was considered as related with cancer.

[c] One case of tension headache was considered as related with cancer.

Table S3. Socio-demographics characteristics of the participants, lifestyles, and co-morbidities, at the baseline evaluation, before any treatment, according to the presence of neuropathic pain (NP) over the five years of follow-up – participants who presented NP in at least one evaluation *vs.* participants who never had NP.

	Patients who never had NP n (%)	Patients with NP at least once n (%)	P value
Living in Greater Porto Area			0.636
No	168 (56.0)	87 (53.7)	
Yes	132 (44.0)	75 (46.3)	
Marital status			**0.044**
Married/living together	200 (66.7)	123 (75.9)	
Single	39 (13.0)	10 (6.2)	
Widower/divorced	61 (20.3)	29 (17.9)	
Professionally active			0.086
No	150 (50.3)	68 (42.0)	
Yes	148 (49.7)	94 (58.0)	
Monthly income above EUR 500 [a]			0.075
No	154 (52.0)	96 (60.8)	
Yes	142 (48.0)	62 (39.2)	
Alcohol consumption, more than 10g/day			0.782
No	239 (79.7)	130 (80.7)	
Yes	61 (20.3)	31 (19.3)	
Past or current smoker			0.078
No	245 (81.7)	121 (74.7)	
Yes	55 (18.3)	41 (25.3)	
Daily consumption of fruits and vegetables of at least 5 portions			0.890
No	235 (78.3)	126 (77.8)	
Yes	65 (21.7)	36 (22.2)	
Playing a sport			0.119
No	242 (80.7)	140 (86.4)	
Yes	58 (19.3)	22 (13.6)	
Body Index (kg/m2)			0.925
<18.5	3 (1.0)	2 (1.2)	
18.5-24.9	132 (44.0)	66 (41.0)	
25.0-29.9	100 (33.3)	55 (34.2)	
≥ 30.0	65 (21.7)	38 (23.6)	
Comorbidities			
Hypertension			0.276
No	200 (66.7)	116 (71.6)	
Yes	100 (33.3)	46 (28.4)	
Diabetes			0.713
No	269 (89.7)	147 (90.7)	
Yes	31 (10.3)	15 (9.3)	
Chronic medicines consumption			0.609
None	106 (35.3)	59 (36.4)	
One	55 (18.3)	22 (13.6)	
Two to four	95 (31.7)	54 (33.3)	
More than four	44 (14.7)	27 (16.7)	

[a] EUR500 is the median value of monthly income in the sample

Table S4. Socio-demographics characteristics of the participants, lifestyles, and co-morbidities, at the baseline evaluation, before any treatment, according to the presence of neuropathic pain (NP) over the five years of follow-up – participants who presented NP at the five-year evaluation *vs.* participants who never had NP.

	Patients who never had NP n (%)	Patients with NP at five years n (%)	P value
Living in Greater Porto Area			0.300
No	168 (56.0)	37 (49.3)	
Yes	132 (44.0)	38 (50.7)	
Marital status			0.849
Married/living together	200 (66.7)	52 (69.3)	
Single	39 (13.0)	8 (10.7)	
Widower/divorced	61 (20.3)	15 (20.0)	
Professionally active			0.439
No	150 (50.3)	34 (45.3)	
Yes	148 (49.7)	41 (54.7)	
Monthly income above EUR 500 [a]			0.206
No	154 (52.0)	44 (60.3)	
Yes	142 (48.0)	29 (39.7)	
Alcohol consumption, more than 10g/day			0.747
No	239 (79.7)	61 (81.3)	
Yes	61 (20.3)	14 (18.7)	
Past or current smoker			0.268
No	245 (81.7)	57 (76.0)	
Yes	55 (18.3)	18 (24.0)	
Daily consumption of fruits and vegetables of at least 5 portions			0.753
No	235 (78.3)	60 (80.0)	
Yes	65 (21.7)	15 (20.0)	
Playing a sport			0.228
No	242 (80.7)	65 (86.7)	
Yes	58 (19.3)	10 (13.3)	
Body Index (kg/m2)			0.811
<18.5	3 (1.0)	1 (1.4)	
18.5-24.9	132 (44.0)	28 (37.8)	
25.0-29.9	100 (33.3)	27 (36.5)	
≥ 30.0	65 (21.7)	18 (24.3)	
Comorbidities			
Hypertension			0.183
No	200 (66.7)	56 (74.7)	
Yes	100 (33.3)	19 (25.3)	
Diabetes			0.797
No	269 (89.7)	68 (90.7)	
Yes	31 (10.3)	7 (9.3)	
Chronic medicines consumption			0.282
None	106 (35.3)	27 (36.0)	
One	55 (18.3)	7 (9.3)	
Two to four	95 (31.7)	28 (37.3)	
More than four	44 (14.7)	13 (17.3)	

[a] EUR500 is the median value of monthly income in the sample

Table S5. Socio-demographics characteristics of the participants, lifestyles, and co-morbidities, at the baseline evaluation, before any treatment, according to the presence of neuropathic pain (NP) over the five years of follow-up – participants who presented persistently NP in all evaluations *vs.* participants who never had NP.

	Patients who never had NP n (%)	Patients with NP in all evaluations n (%)	P value
Living in Greater Porto Area			0.713
No	168 (56.0)	19 (52.8)	
Yes	132 (44.0)	17 (47.2)	
Marital status			0.933
Married/living together	200 (66.7)	25 (69.4)	
Single	39 (13.0)	4 (11.1)	
Widower/divorced	61 (20.3)	7 (19.4)	
Professionally active			0.504
No	150 (50.3)	16 (44.4)	
Yes	148 (49.7)	20 (55.6)	
Monthly income above EUR 500 [a]			0.567
No	154 (52.0)	20 (57.1)	
Yes	142 (48.0)	15 (42.9)	
Alcohol consumption, more than 10g/day			0.603
No	239 (79.7)	30 (83.3)	
Yes	61 (20.3)	6 (16.7)	
Past or current smoker			0.871
No	245 (81.7)	29 (80.6)	
Yes	55 (18.3)	7 (19.4)	
Daily consumption of fruits and vegetables of at least 5 portions			0.228
No	235 (78.3)	25 (69.4)	
Yes	65 (21.7)	11 (30.6)	
Playing a sport			0.106
No	242 (80.7)	33 (91.7)	
Yes	58 (19.3)	3 (8.3)	
Body Index (kg/m2)			0.460
<18.5	3 (1.0)	0 (0.0)	
18.5-24.9	132 (44.0)	11 (31.4)	
25.0-29.9	100 (33.3)	14 (40.0)	
≥ 30.0	65 (21.7)	10 (28.6)	
Comorbidities			
Hypertension			0.091
No	200 (66.7)	29 (80.6)	
Yes	100 (33.3)	7 (19.4)	
Diabetes			0.885
No	269 (89.7)	32 (88.9)	
Yes	31 (10.3)	4 (11.1)	
Chronic medicines consumption			0.643
None	106 (35.3)	12 (33.3)	
One	55 (18.3)	4 (11.1)	
Two to four	95 (31.7)	13 (36.1)	
More than four	44 (14.7)	7 (19.4)	

[a] EUR500 is the median value of monthly income in the sample

Table S6. Socio-demographics characteristics of the participants, lifestyles, and co-morbidities, at the baseline evaluation, before any treatment, according to the presence of chemotherapy-induced peripheral neuropathy (CIPN) over the five years of follow-up among those who were treated with chemotherapy– participants who presented CIPN in at least one evaluation *vs.* participants who never had CIPN.

	Patients who never had CIPN n (%)	Patients with CIPN at least once n (%)	P value
Living in Greater Porto Area			0.172
No	117 (56.5)	35 (47.3)	
Yes	90 (43.5)	39 (52.7)	
Marital status			0.901
Married/living together	147 (71.0)	51 (68.9)	
Single	23 (11.1)	8 (10.8)	
Widower/divorced	37 (17.9)	15 (20.3)	
Professionally active			0.531
No	86 (41.7)	34 (45.9)	
Yes	120 (58.3)	40 (54.1)	
Monthly income above EUR 500 [a]			0.914
No	109 (53.4)	39 (54.2)	
Yes	95 (46.6)	33 (45.8)	
Alcohol consumption, more than 10g/day			0.869
No	41 (19.8)	14 (18.9)	
Yes	166 (80.2)	60 (81.1)	
Past or current smoker			0.434
No	163 (78.7)	55 (74.3)	
Yes	44 (21.3)	19 (25.7)	
Daily consumption of fruits and vegetables of at least 5 portions			0.186
No	155 (74.9)	61 (82.4)	
Yes	52 (25.1)	13 (17.6)	
Playing a sport			0.497
No	169 (81.6)	63 (85.1)	
Yes	38 (18.4)	11 (14.9)	
Body Index (kg/m2)			0.396
<18.5	3 (1.4)	0 (0.0)	
18.5-24.9	83 (40.1)	30 (41.1)	
25.0-29.9	73 (35.3)	31 (42.5)	
≥ 30.0	48 (23.2)	12 (16.4)	
Comorbidities			0.957
Hypertension	156 (75.4)	56 (75.7)	
No	51 (24.6)	18 (24.3)	
Yes			0.232
Diabetes	190 (91.8)	71 (95.9)	
No	17 (8.2)	3 (4.1)	
Yes			
Chronic medicines consumption			0.282
None	83 (40.1)	30 (40.5)	
One	42 (20.3)	8 (10.8)	
Two to four	61 (29.5)	27 (36.5)	
More than four	21 (10.1)	9 (12.2)	

[a] EUR500 is the median value of monthly income in the sample

Table S7. Socio-demographics characteristics of the participants, lifestyles, and co-morbidities, at the baseline evaluation, before any treatment, according to the presence of chemotherapy-induced peripheral neuropathy (CIPN) over the five years of follow-up among those who were treated with chemotherapy– participants who presented CIPN at the five-year evaluation *vs.* participants who never had CIPN.

	Patients who never had CIPN n (%)	Patients with CIPN at all evaluations n (%)	P value
Living in Greater Porto Area			0.081
No	117 (56.5)	19 (42.2)	
Yes	90 (43.5)	26 (57.8)	
Marital status			0.789
Married/living together	147 (71.0)	30 (66.7)	
Single	23 (11.1)	5 (11.1)	
Widower/divorced	37 (17.9)	10 (22.2)	
Professionally active			0.740
No	86 (41.7)	20 (44.4)	
Yes	120 (58.3)	25 (55.6)	
Monthly income above EUR 500 [a]			0.492
No	109 (53.4)	21 (47.7)	
Yes	95 (46.6)	23 (52.3)	
Alcohol consumption, more than 10g/day			0.715
No	41 (19.8)	10 (22.2)	
Yes	166 (80.2)	35 (77.8)	
Past or current smoker			0.886
No	163 (78.7)	35 (77.8)	
Yes	44 (21.3)	10 (22.2)	
Daily consumption of fruits and vegetables of at least 5 portions			0.170
No	155 (74.9)	38 (84.4)	
Yes	52 (25.1)	7 (15.6)	
Playing a sport			0.798
No	169 (81.6)	36 (80.0)	
Yes	38 (18.4)	9 (20.0)	
Body Index (kg/m2)			0.726
<18.5	3 (1.4)	0 (0.0)	
18.5-24.9	83 (40.1)	20 (45.5)	
25.0-29.9	73 (35.3)	16 (36.4)	
≥ 30.0	48 (23.2)	8 (18.2)	
Comorbidities			
Hypertension			0.508
No	156 (75.4)	36 (80.0)	
Yes	51 (24.6)	9 (20.0)	
Diabetes			0.386
No	190 (91.8)	43 (95.6)	
Yes	17 (8.2)	2 (4.4)	
Chronic medicines consumption			0.485
None	83 (40.1)	22 (48.9)	
One	42 (20.3)	5 (11.1)	
Two to four	61 (29.5)	14 (31.1)	
More than four	21 (10.1)	4 (8.9)	

[a] EUR500 is the median value of monthly income in the sample

Table S8. Socio-demographics characteristics of the participants, lifestyles, and co-morbidities, at the baseline evaluation, before any treatment, according to the presence of chemotherapy-induced peripheral neuropathy (CIPN) over the five years of follow-up among those who were treated with chemotherapy– participants who presented CIPN at all evaluations *vs.* participants who never had CIPN.

	Patients who never had NP n (%)	Patients with NP in all evaluations n (%)	P value
Living in Greater Porto Area			0.388
No	168 (56.0)	16 (48.5)	
Yes	132 (44.0)	17 (51.5)	
Marital status			0.871
Married/living together	200 (66.7)	22 (66.7)	
Single	39 (13.0)	4 (12.1)	
Widower/divorced	61 (20.3)	7 (21.2)	
Professionally active			0.942
No	150 (50.3)	14 (42.4)	
Yes	148 (49.7)	19 (57.6)	
Monthly income above EUR 500 [a]			0.718
No	154 (52.0)	16 (50.0)	
Yes	142 (48.0)	16 (50.0)	
Alcohol consumption, more than 10g/day			0.327
No	239 (79.7)	9 (27.3)	
Yes	61 (20.3)	24 (72.7)	
Past or current smoker			0.439
No	245 (81.7)	24 (72.7)	
Yes	55 (18.3)	9 (27.3)	
Daily consumption of fruits and vegetables of at least 5 portions			**0.042**
No	235 (78.3)	30 (90.9)	
Yes	65 (21.7)	3 (9.1)	
Playing a sport			0.656
No	242 (80.7)	28 (84.8)	
Yes	58 (19.3)	5 (15.2)	
Body Index (kg/m2)			0.753
<18.5	3 (1.0)	0 (0.0)	
18.5-24.9	132 (44.0)	13 (39.4)	
25.0-29.9	100 (33.3)	14 (42.4)	
≥ 30.0	65 (21.7)	6 (18.2)	
Comorbidities			
Hypertension			0.418
No	200 (66.7)	27 (81.8)	
Yes	100 (33.3)	6 (18.2)	
Diabetes			0.671
No	269 (89.7)	31 (93.9)	
Yes	31 (10.3)	2 (6.1)	
Chronic medicines consumption			0.546
None	106 (35.3)	15 (45.5)	
One	55 (18.3)	4 (12.1)	
Two to four	95 (31.7)	12 (36.4)	
More than four	44 (14.7)	2 (6.1)	

[a] EUR500 is the median value of monthly income in the sample

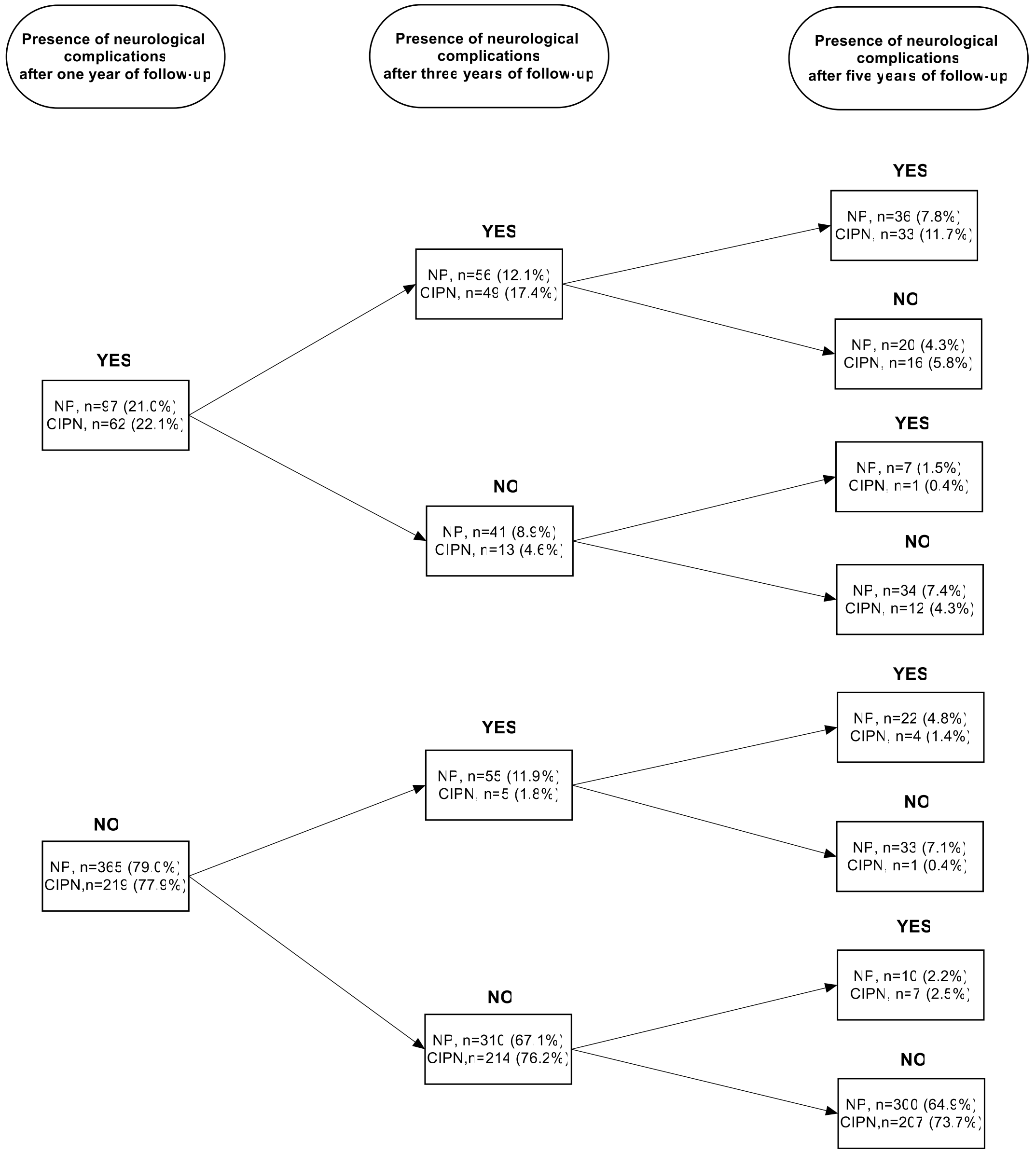

Figure S1. Presence of neurological complications after one, three and five years after diagnosis.
NP, Neuropathic pain; CIPN, Chemotherapy-induced peripheral neuropathy.
For neuropathic pain, the percentages are relative to the entire sample, N=462; for chemotherapy-induced peripheral neuropayhy, the percentages are relative to participants who were treated with chemotherapy over the five years, N=281.

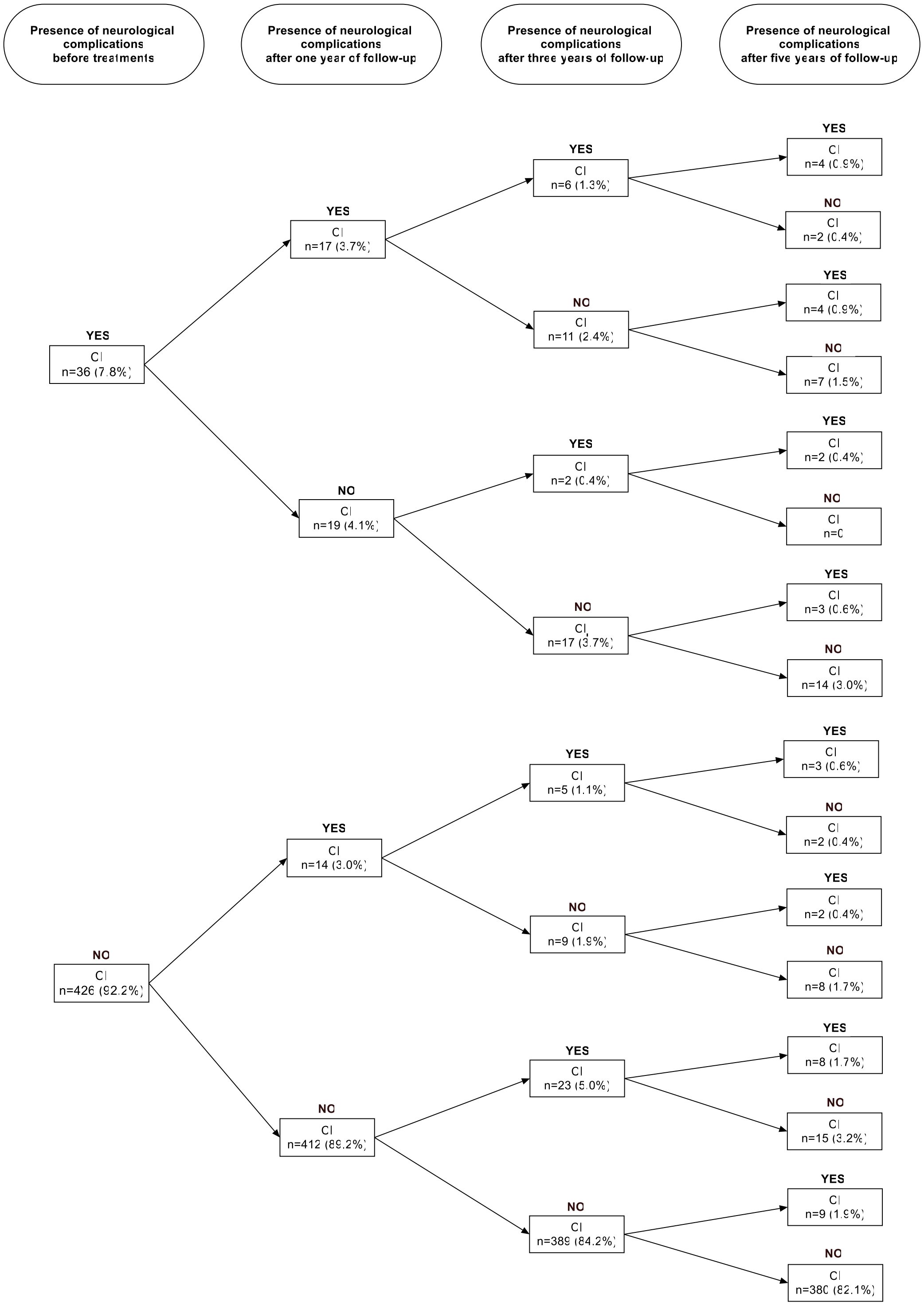

Figure S2. Presence of cognitive impairment before treatments and at one, three and five years since diagnosis. CI, cognitive impairment.

The percentages are relative to the entire sample number, N=462.
